# Effective surveillance of acute COVID-19 using a cost- and labor-efficient approach: a paradigm for the longitudinal monitoring of respiratory infections in larger cohorts

**DOI:** 10.1007/s15010-025-02526-8

**Published:** 2025-05-12

**Authors:** Paul R. Wratil, Niklas A. Schmacke, Burak Karakoc, Christopher Dächert, Elif Apak, Franziska Krenn, Sara Bjedov, Irina Badell, Tamara Pflantz, Alexandra Lübke, Vanessa Ferrari, Aldric Namias, Alexander Graf, Natascha Grzimek-Koschewa, Helga Mairhofer, Ina Koeva-Slancheva, Stefan Hörmansdorfer, Stefan Krebs, Helmut Blum, Ernst-W. Raschner, Matthias Klein, Stephan Boehm, Veit Hornung, Martin R. Fischer, Oliver T. Keppler

**Affiliations:** 1 Faculty of Medicine, Max von Pettenkofer Institute & Gene Center, LMU München, Pettenkoferstr. 9a, Virology, Munich, 80336 Germany; 2 German Center for Infection Research (DZIF), Partner Site Munich, Munich, Germany; 3 Gene Center, Department of Biochemistry, Faculty of Chemistry and Pharmacy, LMU München, Feodor-Lynen-Str. 25, Munich, 81377 Germany; 4 Dekanat der Medizinischen Fakultät, LMU Klinikum, LMU München, Munich, Germany; 5 Laboratory for Functional Genome Analysis, Gene Center, LMU München, Munich, Germany; 6 Bavarian Health and Food Safety Authority, Oberschleissheim, Germany; 7 Emergency Department, Department of Neurology, LMU Klinikum, LMU München, Munich, Germany; 8 Institute of Medical Education, LMU Klinikum, LMU München, Munich, Germany

**Keywords:** SARS-CoV-2, COVID-19, Surveillance, PCR test, Variant analysis, Longitudinal cohort study

## Abstract

**Purpose:**

To reduce the risk of viral transmission in a large cohort of individuals by longitudinal surveillance of COVID-19.

**Methods:**

A cost- and labor-effective method was developed for longitudinal screening of acute COVID-19 in larger cohorts with high-level data protection. Herein, individuals would submit self-sampled tongue swabs that were analyzed for viral RNA by pooled reverse transcription-polymerase chain reaction (PCR). Results were communicated online and by telephone. Utilizing this workflow, medical and dental students at a quaternary care hospital were regularly tested between December 16, 2020, and February 17, 2023. Virus variant analysis was performed by melting curve PCR and next-generation sequencing.

**Results:**

Our method led to a cost reduction for PCR testing that was greater than 10-fold without compromising the time to result. 3,693 individuals participated, contributing 52,993 samples. 430 cases of acute COVID-19 were detected in total. The testing behavior among participants differed from that of the general population. Periods with high numbers of newly detected cases in the study cohort coincided with high COVID-19 incidences in the public. Furthermore, one COVID-19 outbreak was observed in the cohort that was not matched by an increased incidence in the general population. Longitudinal virus variant analysis showed an overlap between variants detected in the study cohort and the public.

**Conclusion:**

Our method enables cost-effective, longitudinal screening for COVID-19 and possibly other respiratory diseases in larger cohorts. At times of high disease burden or if public surveillance is less vigorous, this approach might be useful for the surveillance of vulnerable individuals and healthcare professionals.

**Supplementary Information:**

The online version contains supplementary material available at 10.1007/s15010-025-02526-8.

## Introduction

The coronavirus disease 2019 (COVID-19) pandemic caused by the viral pathogen severe acute respiratory syndrome coronavirus 2 (SARS‑CoV‑2) led, since its inception in early 2020, to several million deaths and countless cases of severe disease [1]. Today, in early 2025, the burden of COVID-19 on global and national health is less pronounced. Nonetheless, new virus variants with increased virulence and transmissibility might emerge in the future, potentially exacerbating the pandemic [2]. Thus, surveillance of SARS-CoV-2 infections remains important to detect and counteract COVID-19 outbreaks, enable swift adaptation of containment measures, as well as identify the emergence and characterize the risk profile of novel virus variants. Moreover, effective COVID-19 surveillance may be readily modified to monitor infections with other respiratory viruses, including influenza and respiratory syncytial virus. Adequate surveillance, however, requires the availability of optimized tools and methodology to screen for acute infections with SARS-CoV-2 and other viral pathogens in larger cohorts.

Normally, individuals are tested for acute COVID-19 upon displaying typical symptoms or reporting close contacts to SARS-CoV-2-infected persons. In addition, certain populations may be screened for acute COVID-19 routinely, independently of symptoms or reported exposure. Using screening as a surveillance technique, SARS-CoV-2 infections can potentially be detected prior to the development of symptoms and in asymptomatic individuals, who account for approximately one third of all COVID-19 cases [3–7]. Thereby, disease outbreaks may be contained in their earliest stages. During times of high COVID-19 incidence, screening is particularly important in populations that are at risk for severe COVID-19 (e.g., immunocompromised persons) as well as in healthcare workers, who are regularly in close contact with vulnerable individuals.

Rapid tests that detect viral nucleocapsid antigen in nasopharyngeal swab samples are frequently utilized when screening for acute SARS-CoV-2 infections [8–10]. These tests are easy to use, comparably inexpensive, and have a short time to result of only 15–30 min. However, rapid antigen tests commonly have low diagnostic sensitivity, especially in asymptomatic COVID-19 patients and in case of infection with SARS-CoV-2 omicron variants, challenging the rationale of screening with these assays in critical settings altogether [11–15]. Reverse transcription of viral RNA followed by nucleic acid amplification (PCR test) can be utilized to detect even low viral loads in respiratory swab samples (limit of detection < 1,000 copies/mL) and, thus, has a superior diagnostic sensitivity for the detection of acute SARS-CoV-2 infections [16, 17]. Furthermore, high-resolution probe-based melting curve and multiplex-based PCR assays allow for the differentiation between SARS-CoV-2 variants [18]. Major caveats of PCR tests for COVID-19 surveillance, however, are that they need to be performed in a well-equipped laboratory by trained personnel, and their long time to result of at least several hours. These drawbacks make PCR testing considerably more expensive compared to rapid antigen testing and, further, logistically more complicated to perform. The costs for materials, equipment and personnel required for PCR testing can be reduced by optimizing the sampling as well as diagnostic workflows and by pooling samples from several individuals in the same initial PCR reaction. Effectively incorporating pooled PCR testing and virus variant analysis into active surveillance of SARS-CoV-2 infections is, however, challenging.

Developing and refining surveillance and screening approaches for infectious diseases is essential to combat epidemics as well as pandemics and may save lives. This study had two objectives: First, to develop a longitudinal screening strategy for acute COVID-19 based on self-sampling on demand followed by pooled PCR testing. This strategy should include subsequent virus variant analysis in infected individuals via probe-based melting curve PCR and next-generation sequencing. Second, to employ this strategy for the surveillance of a cohort of medical and dental students at the LMU Klinikum, the second largest university hospital in Germany. PCR testing rates, newly detected COVID-19 cases, larger disease outbreaks, and SARS-CoV-2 variants identified among the study participants in a period of more than two years were compared to those in the general population of Bavaria, Germany.

## Materials and methods

### Study design, setting and participants

The study was performed at the hospital of the Ludwig Maximilian University of Munich (LMU Klinikum), Germany. Between December 16, 2020, and September 30, 2021, medical and dental students with patient-related teaching and learning formats in their curriculum and between October 1, 2021, and February 17, 2023, all medical and dental students were invited to participate in this longitudinal study.

### Data collection

Participants could contribute samples on demand but were generally recommended to donate a self-sampled tongue swab for pooled PCR testing every two weeks between December 16, 2020, and September 30, 2021. From October 1, 2021, onwards, participants were encouraged to contribute a sample once per week. Sample donation was possible Monday to Friday between 7.30 am and 6.30 pm.

Throughout the entire study period, individuals in Bavaria were sampled for COVID-19 PCR testing by trained personnel in hospitals and clinics as well as in test centers if they experienced symptoms, presented a positive COVID-19 rapid antigen test, or upon appointment by a physician [19]. Moreover, free-of-charge PCR testing was available without having to specify a reason for the entire population of Bavaria until July 1, 2021 [20]. Time-resolved data on PCR tests performed and newly detected COVID-19 cases among the general population in the state of Bavaria, Germany, were extracted from the Database of the Bavarian Health and Food Safety Authority (Bayerisches Landesamt für Gesundheit und Lebensmittelsicherheit, LGL). Data on SARS-CoV-2 variants detected among COVID-19 cases in Bavaria were drawn from the databases of the Robert Koch Institute (https://github.com/robert-koch-institut/) and the Bavarian Network for the Molecular Genetic Surveillance of SARS-CoV-2 (Bay-VOC, https://www.bay-voc.lmu.de/surveillance.xhtml).

### Detection of SARS-CoV-2 infections using pooled PCR

100 µL of the approximately 1000 µL from each swab sample of eight different students were pooled using an automated liquid handler (Biomek i5 Span-8, Beckman Coulter, USA). Pools were either directly subjected to the quantitative Cobas SARS-CoV-2 test (Roche Diagnostics, Switzerland) on a Cobas 6800 instrument (Roche Diagnostics, Switzerland) or further processed for RNA extraction on an automated liquid handler (Biomek i5 Span-8, Beckman Coulter, USA) using the RNAdvance Viral Reagent Kit (Beckman Coulter, USA). Subsequently, eluates from the RNA extraction were analyzed by reverse transcription quantitative real-time PCR (RT-qPCR) using the either the Allpex 2019 nCoV Assay (Seegene, South Korea) on a BioRad CFX 96 Dx (BioRad, USA) or a self-developed PCR system based on the CDC N1 PCR using the Quantinova Multiplex RT-PCR kit (Qiagen, Germany) on a LightCycler 480 II (Roche Diagnostics, Switzerland). For absolute quantification, standard curves were generated using a dilution series of samples with known concentrations (INSTAND, Germany). If a pooled PCR test was positive, each of the samples contributing to that specimen pool were analyzed individually utilizing the same RNA extraction and RT-qPCR methods as described above to identify which contributor(s) to the pool had a SARS-CoV-2 infection.

### SARS-CoV-2 variant analysis by melting curve PCR and next-generation sequencing

Melting curve analyses were performed as described [18]. Briefly, extracted RNA from SARS-CoV-2 positive specimens was analyzed on a LightCycler 480 II (Roche Diagnostics, Switzerland) with the Luna Probe One-Step RT-qPCR kit (No ROX, NEB, USA) and various VirSNiP kits (TIB MOLBIOL, Germany) chosen according to the most prevalent SARS-CoV-2 variants at the time of swab sampling.

Genotyping by next-generation sequencing was performed as previously described [13, 18]. Detected SARS-CoV-2 variants and subvariants were grouped into the following lineages: alpha, beta, gamma, delta, omicron BA.1/BA.2, BA.4/BA.5, BQ.1, BF.7, XBB.1, and early virus variants that emerged prior to SARS-CoV-2 VoC alpha (denoted as ‘others’).

### Sample and data logistics

We developed a cost- and labor-efficient, privacy-first strategy for sample and data management that we designed to be easy to use for participants without compromising the safety of their personal data.

**Fig. 1:**
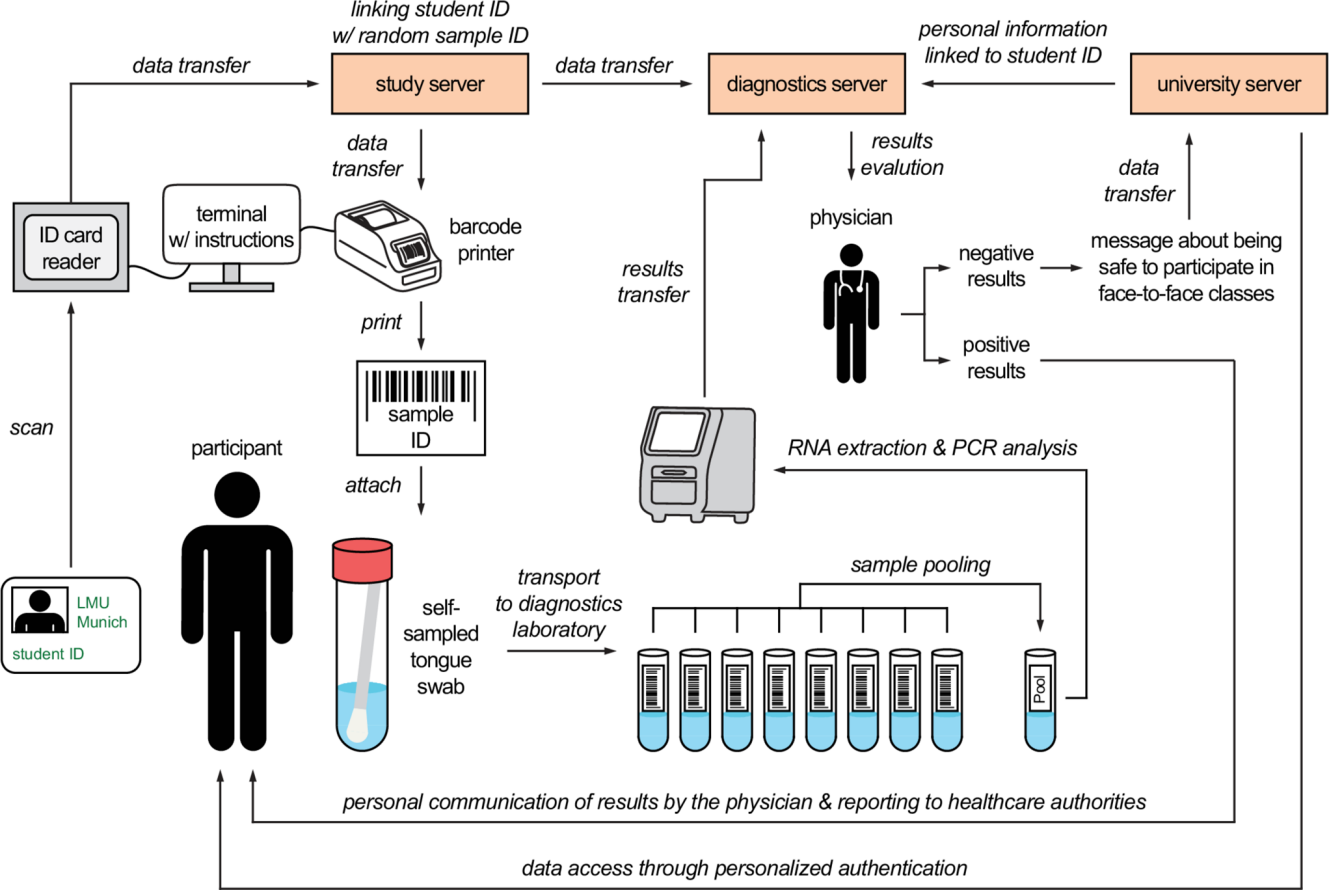
Workflow of the sample and data logistics. To submit a sample, students authenticated at a testing terminal by scanning their student ID card. They were then presented with instructions for the collection of a tongue swab on the terminal’s screen. The terminal printed a label with a barcode encoding a random ID that the study participants attached to their sample tube. Samples were collected and transported to the diagnostics laboratory twice per day. Internally, the random ID was connected to the sampling date and the student ID number read from the student ID card, allowing us to trace samples back to students and sampling times. When test results became available, students were notified of their clearance status for courses where a negative test was acquired. Study participants with positive test results were contacted by the physicians

The sample and data logistics workflow is visualized in Fig. 1. For sample submission, students equipped with their student identity (ID) card visited one of the two sample collection points (one collection point was installed at each of the two main campuses of the university hospital). At the collection points there were dispensers with sterile kits for swab sampling. After drawing one of these kits, participants self-sampled a tongue swab according to instructions provided to them on the study website. Each collection point had a study terminal with a card reader and a barcode printer (Supplementary Fig. 1). Following self-sampling, participants scanned their student ID numbers using the card reader. The terminal, subsequently, sent this ID number to the study server, which saved the ID of the participant together with the time of scanning and linked it to a random number that contained no personal information of the participant (sample ID). The sample ID was sent back to the terminal and printed as a barcode on a label, which the participant attached to their self-sampled swab. Finally, the participant placed the swab sample with the barcode label into a probe rack at the collection point.

Twice daily, donated samples were transported from the collection points to the diagnostics laboratory of the Max von Pettenkofer Institute, LMU Munich. Here, sample pooling, RNA extraction and SARS-CoV-2-specific RT-qPCR analysis were performed as described above. The results for each sample ID tested were uploaded to the diagnostics server. For assigning the student IDs to the test results, information on student IDs linked to the sample IDs were transferred from the study server to the diagnostics server. The corresponding student IDs of all samples that tested negative on the same day were uploaded to the university server. Participants could log into the university server with their own personalized authentication. To ensure personal data protection, no diagnostic results were communicated via the university server. Instead, participants saw a message after logging into the university server stating that it was likely safe for them to participate in face-to-face classes.

In case of positive test results, the physicians responsible for testing transferred the additional personal information corresponding to the student ID numbers from the university server to the diagnostics server, including the full name, birthday, address, and phone number of the participant. Afterwards, the responsible physicians contacted the participants by phone and, in case of newly diagnosed SARS-CoV-2 infections, reported the COVID-19 cases to the local health authorities, following government regulations.

Using this strategy, diagnostic results linked to personal information of the participants were exclusively handled and stored on the diagnostics server that was not connected to the internet and protected by additional means from access by third parties, ensuring a high level of safety for the personal data of the participants.

### Statistical analysis

Data were analyzed in Prism version 9.0.1 (GraphPad Software Inc., USA). Pairwise comparisons were tested for their statistical significance using Fisher’s exact test with Holm-Šidák’s multiple testing correction.

## Results

### Attendance and adherence to the study

3,693 individuals enrolled in the study and submitted at least one tongue swab sample. Over the entire course of the study, a total of 52,993 specimens were analyzed for their abundance of SARS-CoV-2-specific RNA by pooled RT-qPCR, i.e., on average 14 samples per participant. The time to result was between 8 h and 30 h, approximately, depending on the time of sample donation.

**Fig. 2:**
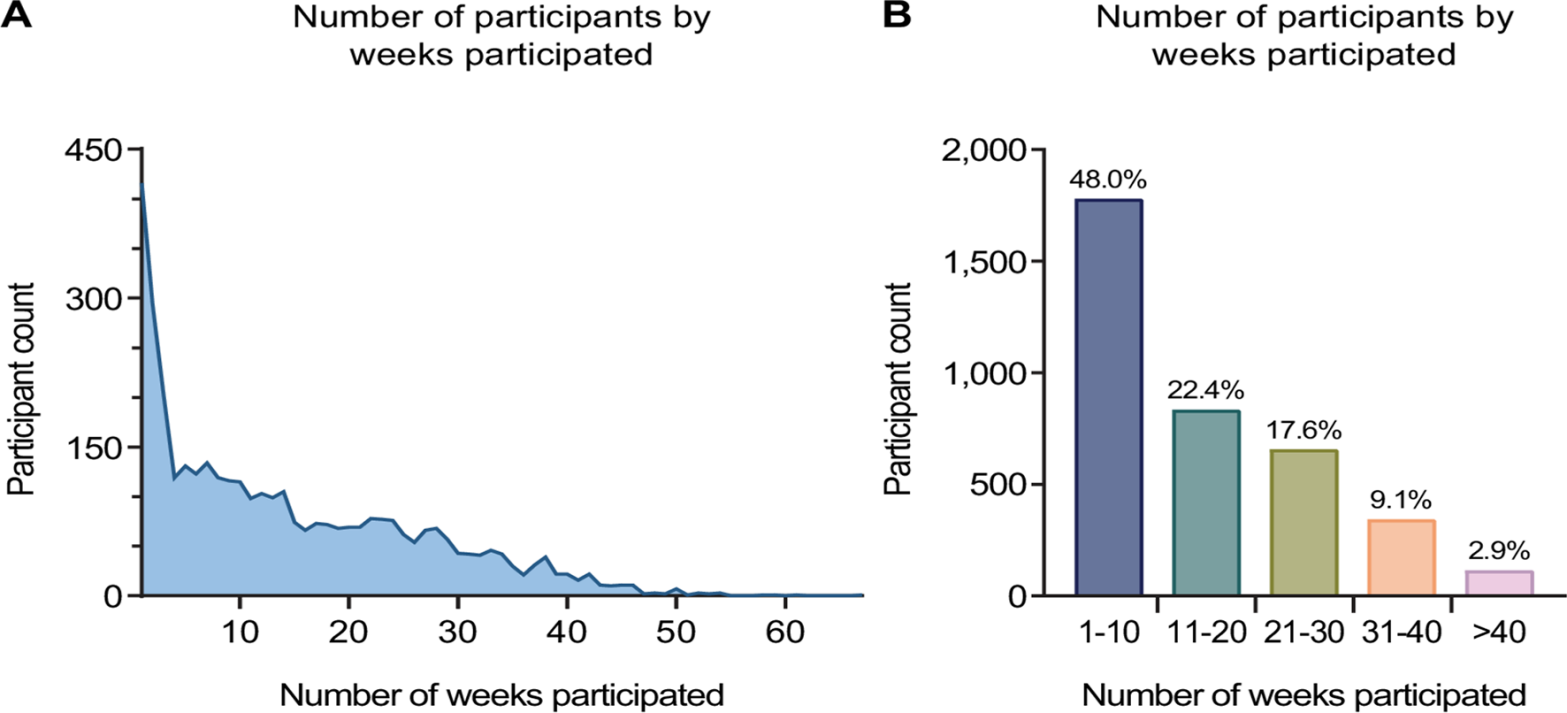
Adherence to the study. (**A**) Number of study participants by the number of weeks they participated in the study. (**B**) Number of participants by the grouped number of weeks they participated in the study. Percentages above bars indicate the fraction of participants having participated a certain number of weeks among all study participants

Samples were collected for a total of 110 weeks. To estimate participants’ adherence, we evaluated the number of weeks in which they submitted a sample. There was a steep decline from the largest fraction of participants who contributed a swab sample only once, to those who participated three times (Fig. 2A). For participants who submitted a sample in more than three separate weeks, we observed less of a decline up to participants who submitted a sample in more than 50 separate weeks (Fig. 2A). Grouping individuals by the number of weeks they submitted swab samples, we found that 48.0% (1,772/3,693) participated one to ten times in the study (Fig. 2B). 22.4% (827/3,693) participated between 11 and 20 times, 17.6% (650/3,693) between 21 and 30 times, and 9.1% (336/3,693) between 31 and 40 times (Fig. 2B). Only a small fraction of individuals, i.e., 2.9% (108/3,693), donated samples more than 40 times (Fig. 2B).

### Longitudinal analysis of tests performed and newly detected COVID-19 cases

**Fig. 3:**
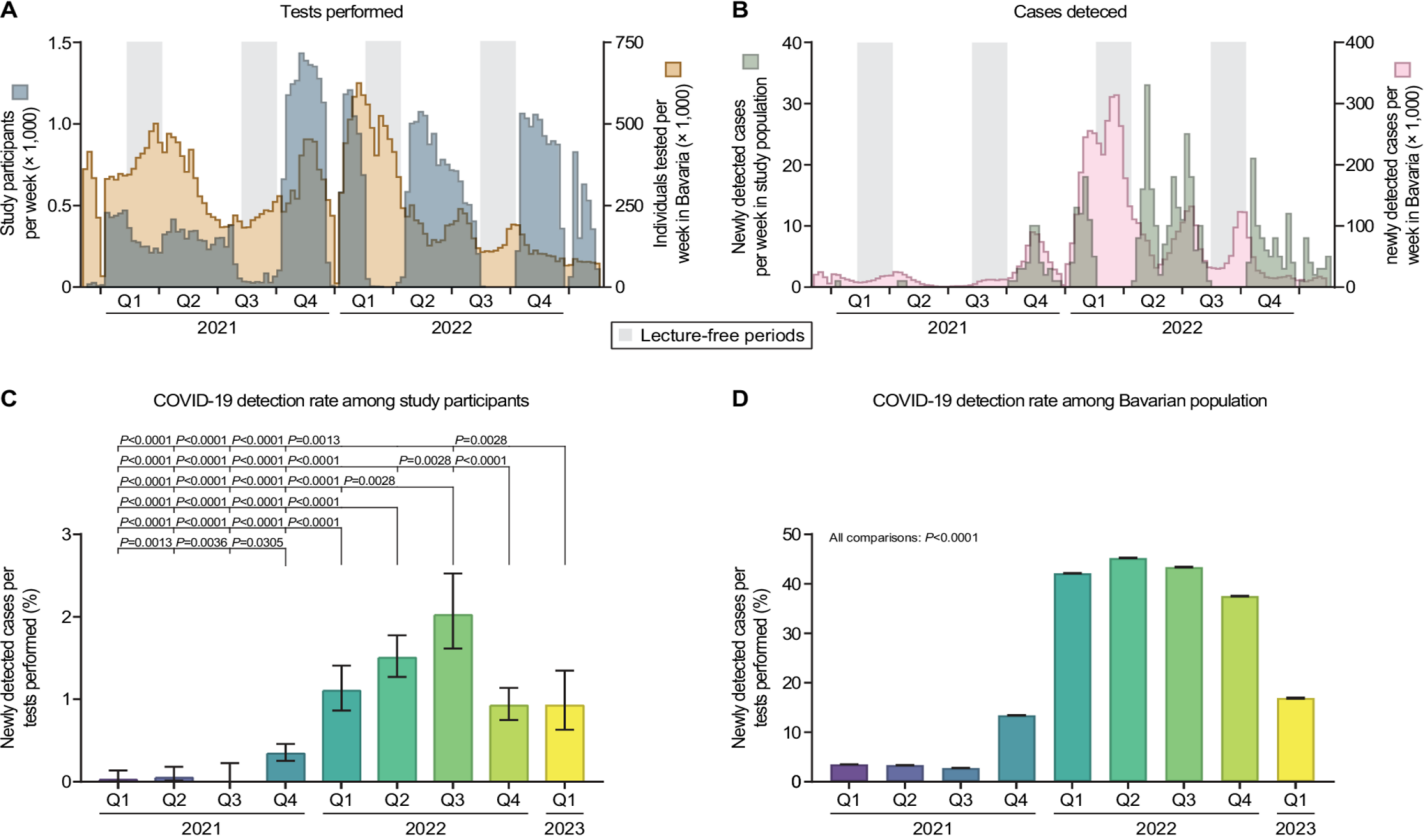
Longitudinal analysis of tests performed and newly detected COVID-19 cases. (**A**) Weekly number of study participants (dark blue) and weekly number of PCR tests for the detection of COVID-19 performed in the Bavarian population (orange) between December 16, 2020, and February 17, 2023. (**B**) In the same period, number of newly detected COVID-19 cases in the study cohort (dark green) and the Bavarian population (pink). (**C**, **D**) Rate of newly detected COVID-19 cases per number of tests performed in every quarter year between January 1, 2021 and February 17, 2023, in the study cohort (**C**) and the public (**D**). Error bars (in **C**, **D**) indicate 95% confidence intervals obtained from Wilson corrected binominal testing. Differences between groups (in **C**, **D**) were tested for their statical significance using Fisher’s exact test with Holm-Šidák’s multiple testing correction. *P*-values are depicted if significant differences were detected. Q– quarter year

We registered on average 482 participants per week with a maximum of 1,433 individuals contributing a tongue swab sample in the week of November 15–22, 2021. Participants submitted specimens for PCR testing mainly at the time when they visited lectures and classes, whereas attendance was low during lecture-free periods and not possible during the Christmas holidays (Fig. 3A, dark blue). The numbers of participants per week increased drastically starting in the fourth quarter of 2021, coinciding with a recommendation for an increased frequency of sample submission from once every two weeks to once per week, and with extending the invitation to participate from students with bedside courses in their curriculum to all students in the medicine and dentistry programs (Fig. 3A, dark blue). The longitudinal testing pattern in the study cohort was strikingly different from that in the general population of Bavaria, Germany (i.e., the site of the study): Among study participants, sample submission numbers were high at the beginning of each term and decreased towards its end (Fig. 3A, dark blue). High numbers of individuals tested by PCR among the Bavarian population (Fig. 3A, orange), on the contrary, were concomitant with the outbreak of new SARS-CoV-2 variants of concern (VoCs) that caused a surge in the overall COVID-19 case numbers (Fig. 3B, pink).

Over the entire study period, a total of 430 acute SARS-CoV-2 infections were discovered among the 3,693 participants. In 21 individuals, we detected two independent infections with at least 4 months between the sampling dates. Regardless of differences in testing patterns, high numbers of newly detected COVID-19 cases in the study cohort (Fig. 3B, dark green) often coincided with high case numbers in the general population (Fig. 3B, pink). This was especially pronounced during pandemic waves dominated by VoC delta in late 2021, VoC omicron BA.1 in January and early February 2022, VoC omicron BA.5 in June and July 2022, and VoCs omicron BA.5 as well as BF.7 in September and October 2022. The highest COVID-19 case numbers in the Bavarian population were observed in March 2022 after the emergence of VoC omicron BA.2 (Fig. 3B, pink). At the same time, there was a lecture-free period that led to low attendance to our study (Fig. 3A, dark blue). The highest numbers of newly detected COVID-19 cases in the study cohort were, however, observed in early May 2022 (Fig. 3B, dark green), when the case numbers in the Bavarian population (Fig. 3B, pink) were comparatively low.

Before the major outbreak of VoC omicron BA.1 in the beginning of 2022, we found a moderate yet statistically significant correlation between individuals tested per week and newly detected cases both in the study cohort (Spearman’s *r* = 0.63, *P* < 0.0001) and in the Bavarian population (*r* = 0.58, *P* < 0.0001). From 2022 onwards, we observed a strong correlation between tests administered and case numbers in the study cohort (*r* = 0.80, *P* < 0.0001) and the general population, respectively (*r* = 0.94, *P* < 0.0001). Detecting these correlations prompted us to calculate the rate of newly detected COVID-19 cases per number of tests performed. In every quarter year analyzed, we found this rate to be significantly lower in study participants compared to individuals tested in the general population (all comparisons: *P* < 0.0001). Both in the study cohort and the Bavarian population, the rates of newly detected cases per tests performed were significantly elevated in 2022 and early 2023 compared to 2021 (Fig. 3C, D).

### SARS-CoV-2 variant analysis

**Fig. 4:**
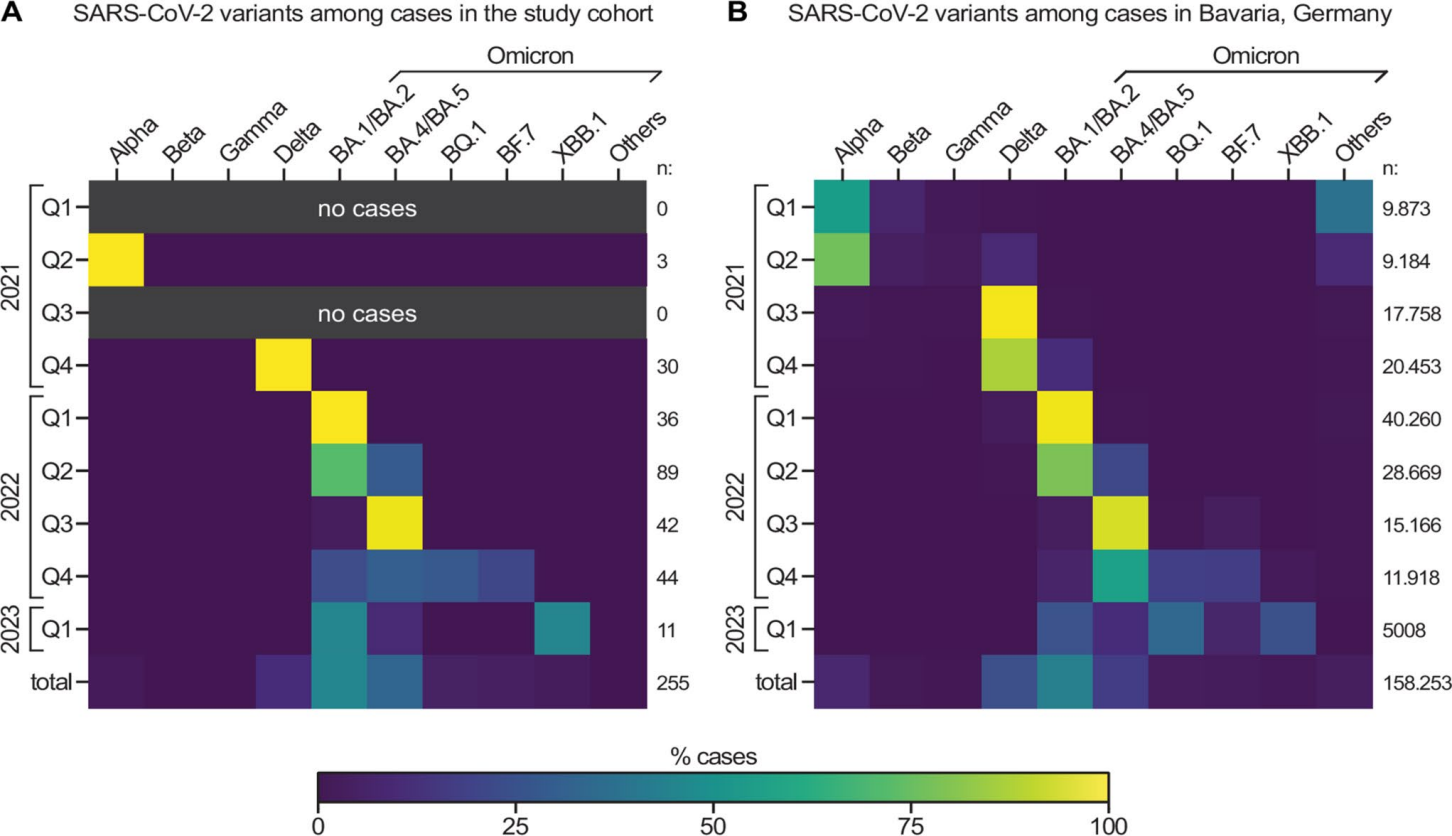
SARS-CoV-2 variant analysis. Heatmaps depicting the percentages of certain SARS-CoV-2 variants detected by melting curve PCR or next generation sequencing in the study cohort (**A**) and the general population of Bavaria (**B**) by quarter year and in total. The number of newly detected COVID-19 cases in each period (n) is shown in (**A**) and (**B**) on the right side of each row

Using melting curve PCR analysis and next-generation sequencing, SARS-CoV-2 variant analysis was successfully performed in 59.3% (255/430) of all COVID-19 cases detected in the study cohort. Evaluating the entire study period, the rates of identified virus variants differed considerably between the study cohort and the Bavarian population (Fig. 4, total): lower rates of SARS-CoV-2 VoCs delta and omicron BA.1/BA.2 were discovered among participants compared to the general population (2.3% vs. 14.7% and 13.2% vs. 42.4%, respectively). Conversely, the rate of omicron BA.4/BA.5 was substantially higher in the study cohort than in the public (62.0% vs. 17.4 ).

We suspected that the observed differences between the study cohort and the general population could be due to comparably low case numbers among participants. Further, our study cohort may have been more likely than the public to contribute positive test results corresponding to asymptomatic SARS-CoV-2 infections or cases with mild disease severity. Additionally, variations in the kinetics of sampling and COVID-19 case rates may have caused these discrepancies. In longitudinal analysis, there was, indeed, a more substantial overlap comparing study participants and the Bavarian population (Fig. 4). To our surprise, however, no infection with Omicron BQ.1 was registered in the study cohort in early 2023, while, at the same time, this variant was the most prevalent in the Bavarian population accounting for 32.7% of all variants identified.

## Discussion

In this study, we established a diagnostic workflow for the longitudinal surveillance of acute SARS-CoV-2 infections and potentially other respiratory viruses by quantitative PCR in large groups of individuals. Due to its sample and data management, as well as due to the use of sample pooling, this workflow is highly cost- and labor-efficient. Simultaneously, its data architecture allows for easy access for participants and physicians without compromising the safety of personal data.

We performed a longitudinal cohort study with our newly established diagnostic workflow. Over a period of more than 2 years, medical and dental students were invited to submit self-sampled tongue swabs for pooled PCR analysis. The attendance to the study was high and the testing behavior among participants differed considerably from that of the general population. However, high COVID-19 incidences in the study cohort were often concomitant with high COVID-19 case numbers in the public. The rates of newly detected COVID-19 cases per tests performed were considerably higher from 2022 onwards compared to pre-2022, coinciding with the emergence of SARS-CoV-2 VoC omicron. Time-resolved analysis of virus variants revealed a great overlap between COVID-19 cases among study participants and the Bavarian population.

The combination of self-sampling, pooled PCR testing and online result communication led to a cost reduction of SARS-CoV-2 testing that we estimated to be more than 10-fold compared to PCR testing in routine diagnostics. Thus, our approach is similarly priced as screening for COVID-19 using rapid antigen tests but, presumably, substantially more sensitive. The time to result in our method was, in most cases, comparable to that of PCR testing in routine diagnostics.

Our diagnostic workflow was built on pre-existing infrastructure: All students had a student ID card with an ID number that could be read by an appropriate device. Moreover, a server with restricted access through personalized authentication that was already being used for coordinating education was employed to communicate test results between physicians and participants. Thus, we anticipate it to be challenging to implement a comparable diagnostic workflow on a populational level. Nonetheless, we expect our approach to be feasible as a screening method in universities, hospitals, and companies, where similar infrastructure as described herein is readily available.

Instead of medical professionals sampling nasopharyngeal swabs from study participants, they self-sampled a tongue swab and submitted it for analysis. On one hand, this considerably decreased the cost and labor intensity of the study, but on the other hand, the diagnostic sensitivity of tongue swabs in PCR testing was shown to be somewhat lower than those of nasopharyngeal swabs [21–23]. Moreover, self-sampling is vulnerable to manipulation by participants, e.g., by submitting tubes without the appropriate sample to be tested for acute COVID-19. In addition, sample pooling might impair the diagnostic sensitivity of the PCR measurement. This effect, however, was shown to be marginal [24–26]. To counteract these potential caveats, our diagnostic workflow can easily be modified, e.g. it may be performed in a setup with nasopharyngeal sampling by healthcare professionals. Furthermore, our workflow can be re-adjusted for the measurement and communication of other diagnostic parameters. This includes infection with other viral pathogens with high transmissibility and substantial risk of causing severe respiratory diseases, e.g., influenza and respiratory syncytial virus. Our workflow can be adapted for the surveillance of these RNA viruses simply by modifying the PCR primers.

We recommended participants to submit samples either every two weeks or in weekly intervals. However, a large number of participants contributed swabs fewer than 10 times. We also observed that participation was more frequent at the beginning of each term than towards its end. Of note, a recently acquired, negative PCR test was obligatory for students to participate in bedside courses. Students who preferred to attend such courses in person, instead of completing them online, thus might have participated in our study sporadically and on demand, rather than self-organizing their own COVID-19 tests, potentially explaining the aforementioned testing patterns.

High numbers of PCR tests in the general population were mirrored by the outbreak of novel virus variants causing considerable waves of the ongoing pandemic. This suggests that the testing behavior of the public differed from that of the study cohort. Conceivably, more individuals got tested at times of high COVID-19 incidence because there were more infected persons with typical symptoms. Further, more persons might have tested positive for an acute SARS-CoV-2 infection using rapid antigen tests, when pandemic waves were at their peak, prompting them to officially verify their antigen test result by PCR.

Regardless of differences in the testing behavior, periods of high COVID-19 incidence in the general population often coincided with high numbers of newly detected SARS-CoV-2 infections in our study cohort. On top of that, we registered one short period of high infection numbers in the study cohort in May 2022 that was not matched by high COVID-19 incidences in the public. This potentially indicates the value of screening efforts to detect larger COVID-19 outbreaks in defined cohorts that are not seen on a populational level.

The observation of higher COVID-19 rates per number of tests being performed in 2022 and 2023, after the emergence of SARS-CoV-2 omicron, possibly points towards the often-reported increased transmissibility of this virus variant that is, partially, due to its pronounced immune escape [27–30]. On July 1, 2021, PCR-testing on demand without the need to provide a reason was replaced in Bavaria by the requirement of a positive rapid antigen test for asymptomatic persons to become PCR tested [19, 20], potentially contributing to the high rates of positive individuals per PCR tests performed in the general population.

After changing the recommendations for sample submission in our study from once every two weeks to once per week in October 2021, we observed a considerable increase in the amount of submitted tongue swab specimens. This may have increased the sensitivity to detect COVID-19 cases in the study cohort. Shortly after changing the submission recommendations, however, COVID-19 incidences in the general population surged dramatically. Therefore, we assume that the increase in newly detected SARS-CoV-2 infections among the study participants after changing the sample submission recommendations was rather due to higher COVID-19 incidences at that time than due to increased sensitivity for the detection of COVID-19 in the cohort.

A limitation of the study is that the attendance of participants dropped considerably during the holiday periods. However, comparing the outbreaks detected in the study with those in the general population showed that no major SARS-CoV-2 outbreak was missed in the study cohort.

There was a considerable overlap between the SARS-CoV-2 variants longitudinally detected in the study cohort and those registered in the Bavarian population. This finding demonstrates that only those virus variants circulated in the study population that were, at that time, also highly prevalent in the public. Discrepancies in the virus variant distribution between the participants and the general population, e.g. in the beginning of 2023, might be due to low case numbers in the study cohort.

Another limitation that may have contributed to the different results comparing the study cohort and the general population is demographic disparity. Being medical and dental students, study participants likely were by average considerably younger and had a higher percentage of females compared to the Bavarian public [31, 32]. We anticipated these medical and dental students to be highly motivated to participate in the study, submit specimens regularly and not cheat during self-sampling. The adherence of individuals from the general population to participate in a similar study may be lower [33].

In summary, our study introduces a new tool for cost- and labor-efficient surveillance of SARS-CoV-2 infections. We show that it is feasible and cost effective to longitudinally screen for acute COVID-19 over several years in a large cohort of individuals utilizing our method. Our screening approach enables the detection of COVID-19 outbreaks in a surveilled group that are independent of high incidences in the population. In certain settings and during times when infection surveillance on a populational level is less vigorous, tools like ours might be especially valuable to monitor SARS-CoV-2 infections in groups of vulnerable individuals and healthcare professionals. Furthermore, our method can be swiftly adapted for screening of other diagnostic parameters, including newly emerging infectious diseases.

## Electronic supplementary material

Below is the link to the electronic supplementary material.

Supplementary Material 1

## Data Availability

The data used and/or analyzed during the current study are available from the corresponding author on reasonable request.
