# Supplementary material for: Effective surveillance of acute COVID-19 using a cost- and labor-efficient approach: a paradigm for the longitudinal monitoring of respiratory infections in larger cohorts
Source: Infection. 2025 May 12;53(5):1953–63. doi: 10.1007/s15010-025-02526-8 (PMC12460580; doi:10.1007/s15010-025-02526-8)

Supplementary Information:

Effective surveillance of acute COVID-19 using a cost- and labor-efficient approach: a paradigm for the longitudinal monitoring of respiratory infections in larger cohorts

Paul R. Wratil, Niklas A. Schmacke, Burak Karakoc, Christopher Dächert, Elif Apak, Franziska Krenn, Sara Bjedov, Irina Badell, Tamara Pflantz, Alexandra Lübke, Vanessa Ferrari, Aldric Namias, Alexander Graf, Natascha Grzimek-Koschewa, Helga Mairhofer, Ina Koeva-Slancheva, Stefan Hörmansdorfer, Stefan Krebs, Helmut Blum, Ernst-W. Raschner, Matthias Klein, Stephan Boehm, Veit Hornung, Martin Fischer, Oliver T. Keppler

Corresponding Authors:

Paul R. Wratil (wratil@mvp.lmu.de)

Niklas A. Schmacke (schmacke@genzentrum.lmu.de)

Oliver T. Keppler (keppler@mvp.lmu.de)

Supplementary Figure Legends

Supplementary Fig. 1. Photograph of the study terminal, identity (ID) card reader and barcode printer.

Supplementary Figure 1

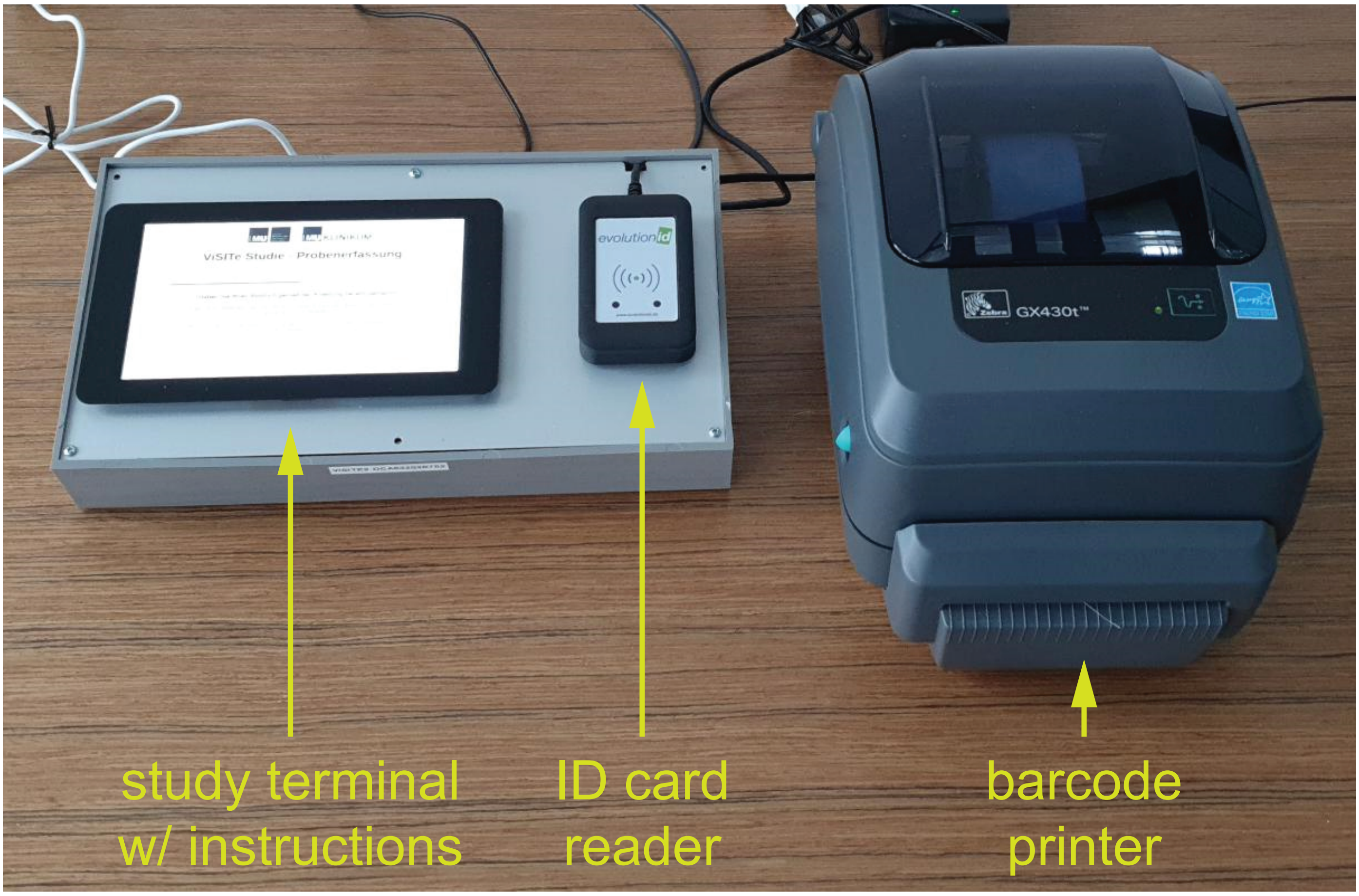

Supplement: Supplementary file 1 — Supplementary Material 1 [file 15010_2025_2526_MOESM1_ESM.pdf]
